# Simplex‐based model for nanoparticle grain identification in four‐dimensional scanning transmission electron microscopy data

**DOI:** 10.1111/jmi.70126

**Published:** 2026-06-03

**Authors:** Wei Liu, Roberto dos Reis, Chad A. Mirkin, Vinayak P. Dravid, Wei Chen, Daniel W. Apley

**Affiliations:** ^1^ Department of Industrial Engineering Northwestern University Evanston Illinois USA; ^2^ Department of Materials Science and Engineering Northwestern University Evanston Illinois USA; ^3^ International Institute for Nanotechnology Northwestern University Evanston Illinois USA; ^4^ Northwestern University Atomic and Nanoscale Characterization Experimental Center Northwestern University Evanston Illinois USA; ^5^ Department of Chemistry Northwestern University Evanston Illinois USA; ^6^ Department of Mechanical Engineering Northwestern University Evanston Illinois USA

**Keywords:** 4D‐STEM segmentation, constrained optimisation, grain identification, polycrystalline materials

## Abstract

Grain identification in polycrystalline nanoparticles, for example, determining which crystal phases are present at each spatial location, is fundamental to materials characterisation. This is particularly challenging when grains overlap extensively, as commonly occurs in four‐dimensional scanning transmission electron microscopy (4D‐STEM) datasets. We propose a simplex‐based model (SBM) in which each simplex vertex represents the diffraction pattern (DP) of a pure grain, and the simplex edges and interior represent overlapping grains. Our SBM grain identification algorithm operates on the Bragg disk (BD) data matrix distilled from the 4D‐STEM data to identify the grain membership at each scan position, together with a BD feature matrix whose columns represent the DPs for each constituent grain, which is important for identifying the crystal structure of each grain. We solve the model using a two‐stage algorithm. In Stage 1, we adapt a linear mixing algorithm to estimate an initial BD feature matrix whose columns represent DPs of potentially overlapping grains. Our Stage 2 algorithm incorporates sparsity considerations to transform the initial BD feature matrix so that its columns represent DPs of pure grains. Using simulated datasets with various grain configurations, we demonstrate that SBM recovers both the BD feature matrix and membership maps more accurately than existing methods, even when a grain lacks any pure region and completely overlaps with other grains.

## 1 INTRODUCTION

Crystalline nanoparticle characterisation is a cornerstone of material discovery, enabling the design of nanomaterials with tailored properties for applications ranging from polymers to catalysts.^1^ Primary characterisation tasks include grain identification or phase mapping to determine the locations and geometry of grains present in a nanoparticle, which is particularly challenging for multi‐element materials.^2^, ^3^ Here ‘grain’ is used as shorthand for nanoparticle regions producing diffraction patterns (DPs) due to different orientations, phases or crystallites. Such techniques involve identifying regions that exhibit distinct DPs due to differences in crystal structure or orientation, which is essential because grain morphology, encompassing shape, size, spatial distribution, and phase composition, directly dictates macroscopic properties including mechanical strength, electrical conductivity, and magnetisation. For example, crystal orientation influences deformation twinning in nanoscale metals that provide desirable properties such as high strength and hardness.^4^

Grain identification based on transmission electron microscopy (TEM) typically relies on the real space image, as TEM provides a DP that is averaged across a selected area that is relatively large (typically at least 10 nm in size but often several hundred nanometres). The emergence of four‐dimensional scanning transmission electron microscopy (4D‐STEM)^5^ has revolutionised nanoparticle characterisation by providing fine lattice information about nanoscale structures. 4D‐STEM records various forms of scattering measurements of a STEM probe in both real and reciprocal spaces, producing a full DP at each real space scan position over a 2D spatial grid covering the nanoparticle. The resulting 4D tensor data contains rich structural information of the material, particularly for crystalline materials where DPs feature clear and periodic Bragg disks (BDs).^6^ However, it is challenging to extract this information buried in the raw 4D‐STEM data, motivating the development of robust statistical models for accurate and efficient characterisation.

Integrating modern statistical tools with crystallographic domain knowledge for 4D‐STEM segmentation remains an open challenge that has stimulated a growing body of research. Thronsen et al. (2024)^7^ benchmarked traditional methods for phase mapping that require substantial prior knowledge (e.g., a library of simulated DPs for known grains) such as template matching and vector matching against machine learning techniques like non‐negative matrix factorisation (NMF) and neural networks over the dataset of an alloy sample. The py4DSTEM framework^8^ includes an algorithm that first groups BDs via the frequency of coexistence of BDs within the same DP, and then segments the nanoparticle image. Other researchers have focused on applying popular segmentation/clustering methods. Shi et al.^9^, ^10^ applied hierarchical clustering and K‐means clustering to segment real space images of 4D‐STEM datasets of ${\mathrm{WS}}_{2}$‐${\mathrm{WSe}}_{2}$ with strain‐engineered structures, which successfully revealed regions of different types/levels of deformations across the sample area. Francis and Voyles (2024)^11^ developed an algorithm built on density‐based spatial clustering that utilises both real space and reciprocal space coordinates of BD centres. Liu et al. (2025)^12^ integrated customised data preprocessing steps with a Gaussian mixture model (GMM) and demonstrated successful results for real datasets. Bruefach et al. (2022)^13^ developed an NMF‐based pipeline, and later specifically investigated the impact of hyperparameter choices on segmentation performance.^14^ NMF has become popular in the field of 4D‐STEM segmentation and has been widely investigated and implemented in other works.^8^, ^15^, ^16^, ^17^ Above works constitute important advances in nanoparticle characterisation, but they are limited by their implicit reliance on the statistical models that underly clustering and NMF algorithms.

This work proposes a simplex‐based model (SBM) that more faithfully represents the nature of nanoparticle 4D‐STEM data, especially when grains overlap. Our SBM algorithm operates on a data feature matrix produced by standard 4D‐STEM data preprocessing tools. Let K denote the number of distinct grains in the nanoparticle, our goal is twofold: (i) recover the pure DP for each of the K distinct grains from the intensity data X (described in Section 2), enabling crystal structure identification, and (ii) determine which grain is present at each scan location i=1,2,…,n, and the degree of membership of the grains (e.g., grain 1 and grain 2 overlap with 30% grain 1 and 70% grain 2), since multiple grains frequently overlap in nanoparticles. Together, this information can be used to construct a 3D representation of the entire nanoparticle that shows the geometry and location of each grain, where they overlap, where they are pure, etc., as well as the pure DP of each grain, even when the grain always overlaps with other grains.

The simplex representation of a nanoparticle embedded in our SBM is crucial for accomplishing this. In Section 2 we define this simplex structure and illustrate its representation of 4D‐STEM data. With this simplex structure as the basis, in Section 3 we present an algorithm for identifying the DPs of the K grains and their memberships at each scan location. This is particularly challenging when some grains are not pure at any scan location (i.e., when a grain overlaps with other grains at every location where it is present). To handle this, we develop a two‐stage algorithm that first estimates a compacted simplex structure and then expands the simplex so that its K vertices represent the DPs of the K pure grains, as opposed to DPs of overlapping grains (see Section 2 for how the simplex vertices relate to the DPs). Section 4 presents numerical results demonstrating that our approach identifies the actual structure of the nanoparticle more accurately than existing methods. Section 5 concludes the paper.

## 2 THE SIMPLEX STRUCTURE OF NANOPARTICLE 4D‐STEM DATA

In this section, we first describe X, and then show how 4D‐STEM data can be compactly represented using a simplex structure. The assumed preprocessing^8^, ^12^ to obtain X involves three main standard steps: (i) identify n scan positions within the nanoparticle and spatially average the DPs locally around each scan position to reduce DP image noise and make the BDs more visible; (ii) detect the BDs in each DP via cross‐correlation (CC) to determine the coordinates of each BD centre and the CC intensity at the centre; (iii) aggregate all detected BD centres across the n DPs to form the Bragg Vector Map (BVM) and compute d local BVM maxima, which are taken to be the set of all distinct detected BD centres across the DPs at all n scan positions. The distilled data features from the preprocessing contain two parts: (i) the collection of coordinates of the d distinct BD centres from the BVM; (ii) the data matrix $X=\left[x_{1},\text{…},x_{n}\right]\in R^{d\times n}$, where each column vector $x_{i}={\left[x_{1,i},\text{…},x_{d,i}\right]}^{T}$ in X is a d‐length vector associated with the ith scan position, and $x_{j,i}$ denotes the CC intensity in the DP for the ith real space scan position at the jth BD centre.

To define a simplex, suppose we have K vectors $b_{0,1},\text{…},b_{0,K}$ (each in $R^{d}$) that represent the featurised DPs for the K distinct crystals in the nanoparticle. That is, $b_{0,k}={\left[b_{0,1,k},\text{…},b_{0,d,k}\right]}^{T}$ is the d‐length vector of intensities at the d BD locations in the BVM, for the pure DP of the kth grain. The K−1 dimensional simplex determined by ${\left\{b_{0,k}\right\}}_{k=1}^{K}$ is defined as the set as

(1) $$\begin{array}{c} C=\{c_{1}b_{0,1}+\cdots +c_{K}b_{0,K}|\sum_{k=1}^{K}c_{k}=1,c_{k}\geq 0,k=1,\text{…},K\}. \end{array}$$

In the context of 4D‐STEM data, we assume at each scan position i, the intensity vector $x_{i}$ at each of the d identified BD locations is a convex linear combination (coefficients that are non‐negative and sum to one) of the BD intensity vectors ${\left\{b_{0,k}\right\}}_{k=1}^{K}$ of the K pure crystal DPs, plus additive noise $\varepsilon_{i}\in R^{d}$. Hence, aside from noise, each $x_{i}$ lies in the simplex C defined in Equation (1), which we can represent compactly as

(2) $$\begin{array}{c} x_{i}=c_{0,1,i}b_{0,1}+\cdots +c_{0,K,i}b_{0,K}+\varepsilon_{i}=\sum_{k=1}^{K}c_{0,k,i}b_{0,k}+\varepsilon_{i}, \end{array}$$

where the membership coefficients ${\left\{c_{0,k,i}\right\}}_{k=1}^{K}$ satisfy the nonnegativity constraint ($c_{0,k,i}\geq 0$ for k=1,…,K) and the sum‐to‐one constraint ($\sum_{k=1}^{K}c_{0,k,i}=1$).

**FIGURE 1:**
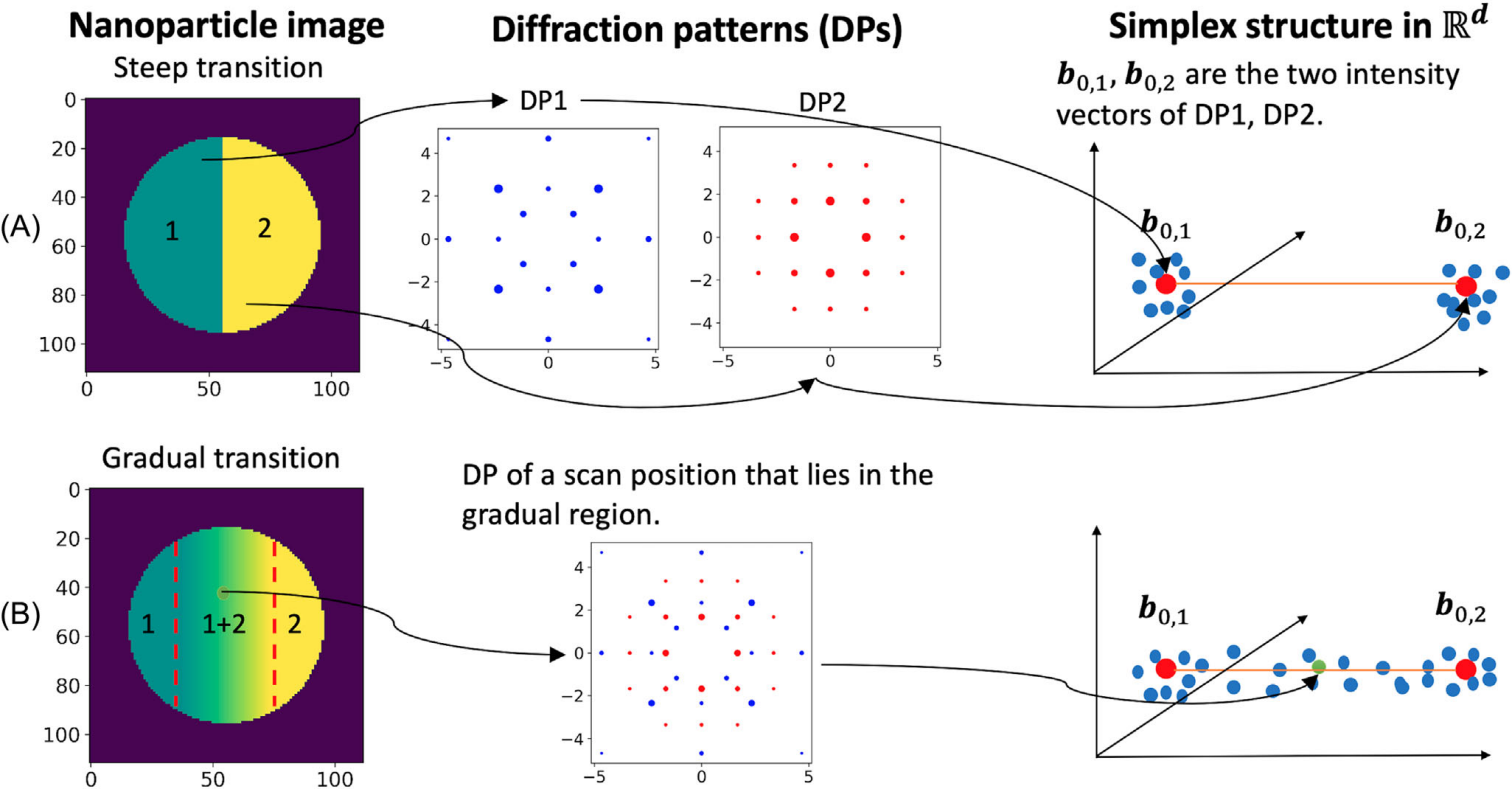
Simplex representation of two‐grain nanoparticles with configurations depicted in the left panels. (A) Steep transition: the nanoparticle (left) contains two pure regions separated by a sharp boundary; each pure region is associated with a distinct DP (middle); in $R^{d}$ space (right), data points (blue dots) cluster near the two vertices $b_{0,1}$ and $b_{0,2}$. (B) Gradual transition: a transition zone (the area between two red dashed lines) produces data points distributed along the simplex edge connecting the vertices. Red and blue dots in the DPs indicate BDs belonging to grains 1 and 2, respectively, and the dot size is proportional to the CC intensities of those BDs. Each blue dot in the simplex structure plot on the right represents the d‐length vector $x_{i}$ at scan position i in the nanoparticle. The arrows indicate the correspondence between scan positions, DPs, and the $R^{d}$ intensity vectors. The axes for nanoparticle images represent pixel coordinates, while the axes for simulated DPs denote the coordinates of BDs.

Figure 1 visualises the simplex structures for two simulated two‐grain nanoparticles, one with a steep transition (no overlap, Figure 1a) between the two grains and the bottom one with a gradual transition (grains overlapping in the middle region, Figure 1b). The two simulated grains are cubic mp‐1425 and cubic mp‐31057 from the Materials Project database,^18^ and their DPs with zone axis [001] are displayed in Figure 1 as DP1 and DP2. For the nanoparticle in Figure 1a, all scan positions on the left of the boundary (labelled region ‘1') share DP1, while those on the right (labelled region ‘2') share DP2. We define a pure region as a contiguous spatial domain where all scan positions correspond to a single crystal grain and therefore have the same DP, and a pure pixel as a pixel within a pure region. Thus, if scan position i is within a pure region for grain k, $c_{0,k,i}=1$, and $c_{0,j,i}=0$ for j≠k. In $R^{d}$ space, the simplex is a line segment connecting two vertices $b_{0,1}$ and $b_{0,2}$ that correspond to DP1 and DP2. Under the noiseless condition, data points for pure pixels would lie exactly at the vertices. In the presence of noise, data points cluster around $b_{0,1}$ and $b_{0,2}$ as in Figure 1a. For the nanoparticle in Figure 1b that is composed of the same two grains but with a gradual transition over which the two grains overlap (the area between two red dashed vertical lines), data points are distributed among the simplex differently. In addition to the clusters around $b_{0,1}$ and $b_{0,2}$ corresponding to the pure regions 1 and 2 in Figure 1b, scan positions in the transition region correspond to points along the line segment connecting $b_{0,1}$ and $b_{0,2}$. For example, the green dot in the nanoparticle image represents a 50/50 balanced mix of the two grains with its DP being the overlap of DP1 and DP2; therefore, in the $R^{d}$ simplex representation, the green dot is located near the centre of the line segment.

**FIGURE 2:**
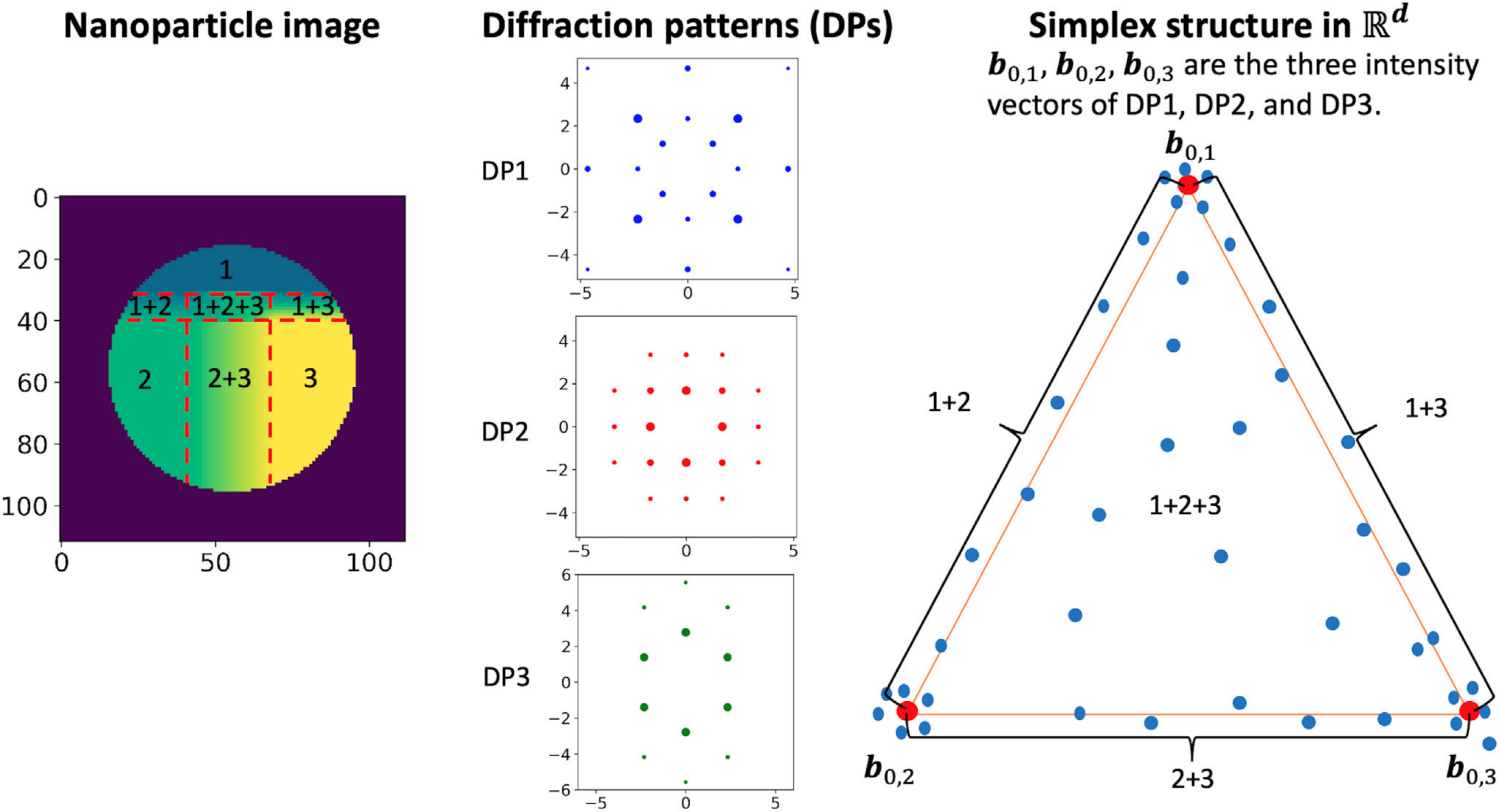
Nanoparticle configuration (left column), example DPs (middle column), and the simplex structure (right column) for the three‐grain nanoparticle. The $R^{d}$ coordinate system is identical to that in Figure 1 but is omitted here and hereafter for visual clarity.

Figure 2 illustrates the simplex for a three‐grain nanoparticle comprised of the same two grains in Figure 1, plus a third grain that is monoclinic mp‐765632. The associated DP with zone axis [001] is displayed in Figure 2 as DP3. The simplex in this case is a two‐dimensional triangle in $R^{d}$. The vertices $b_{0,1}$, $b_{0,2}$, and $b_{0,3}$ represent the pure patterns DP1, DP2, and DP3, respectively. The location of a data point within this simplex reflects the underlying mixture of crystal grains at that scan position: Those associated with pure pixels cluster around vertices, those representing mixtures of two grains lie along the edge connecting the corresponding two vertices, and those for scan positions containing a mixture of all three grains (the region labelled ‘1+2+3') reside within the interior of the simplex. Because the nanoparticle in Figure 2 features a comprehensive set of mixtures, including pure regions for each grain, transition zones between pairs of grains, and three‐grain overlaps, the resulting simplex structure is fully populated with points distributed around the vertices, along the edges, and throughout the interior.

**FIGURE 3:**
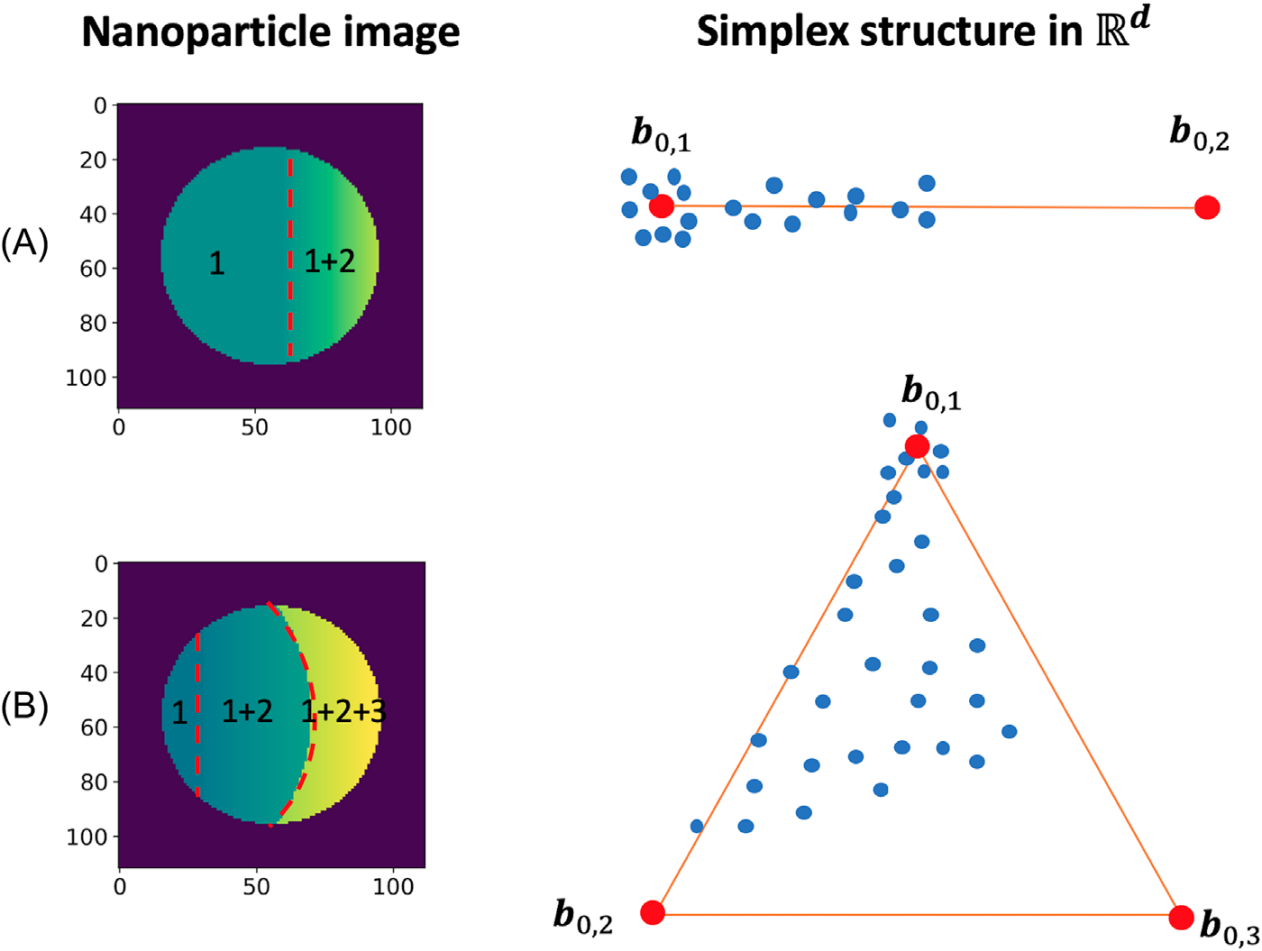
Nanoparticle configurations (left column) and simplex structures (right column) for nanoparticles in which some grains do not have any pure region.

Examples in Figures 1 and 2 are ‘ideal’ because the 4D‐STEM dataset contains pure regions for all K constituent grains. A more challenging and frequently encountered scenario occurs when one or more crystals lack a pure region within the dataset. For example, in the two‐grain nanoparticle in Figure 3a, grain 2 does not have a pure region, with its maximum membership coefficient reaching only around 0.5 at the far right of the nanoparticle. As results in Section 4 show, the absence of pure pixels makes it difficult for NMF to accurately recover the vertex $b_{0,2}$. Similarly, for the three‐grain nanoparticle in Figure 3b, grain 1 is present across the entire nanoparticle so that neither grain 2 nor grain 3 has a pure region. The absence of pure‐grain regions is reflected in the simplex structures shown in the right panels: There are no data points around $b_{0,2}$ in Figure 3a simplex or around $b_{0,2}$ and $b_{0,3}$ in Figure 3b simplex. As results in Section 4 show, because NMF does not fully incorporate the simplex structure, it tends to produce mixtures of ${\left\{b_{0,k}\right\}}_{k=1}^{K}$ as the estimated DPs for the grains, which makes it more challenging to identify the crystal structures of the grains. In contrast, SBM can robustly recover the true DPs even in the absence of pure regions, as demonstrated by simulations in Section 4.

## 3 SBM NANOPARTICLE CHARACTERISATION ALGORITHM

In this section, we first formulate a two‐stage optimisation that we use to fit the SBM to the data X to estimate the simplex vertices that represent the DPs $\left\{b_{0,1}\text{…},b_{0,K}\right\}$ of each grain, as well as the degree of membership of each grain at each scan position. Then, in Sections 3.1 and 3.2, we describe the algorithms that we developed to solve the Stage 1 and Stage 2 optimisations, respectively. Section 3.3 discusses how to select the hyperparameter λ in the Stage 1 optimisation and how to adaptively choose the step size in the optimisation.

Based on Equation (2), the SBM in matrix form can be written as

(3a) $$\begin{array}{cc} & X=B_{0}C_{0}+\varepsilon \end{array}$$

(3b) $$\begin{array}{cc} \text{s.t.}\; & B_{0}\in R_{\geq 0}^{d\times K}, \end{array}$$

(3c) $$\begin{array}{cc} & C_{0}\in R_{\geq 0}^{K\times n},\;C_{0}^{T}1_{K}=1_{n}, \end{array}$$

where $B_{0}=\left[b_{0,1},\text{…},b_{0,K}\right]$ is the matrix of simplex vertices, $C_{0}=\left[\begin{array}{ccc} c_{0,1,1} & \cdots & c_{0,1,n}\\ \vdots & \ddots & \vdots \\ c_{0,K,1} & \cdots & c_{0,K,n} \end{array}\right]$ is the membership matrix whose columns represent the memberships at each scan position i=1,…,n, $R_{\geq 0}^{d\times K}$ denotes the set of d×K matrices with all entries being nonnegative, $1_{K}\in R^{K}$ denotes the K‐length column vector of ones, and $\varepsilon \in R^{d\times n}$ represents the noise matrix. The goal is to estimate $B_{0}$ and $C_{0}$ given X. While K is typically unknown, in Appendix B we describe how to estimate K by performing principal component analysis (PCA) on X.

**FIGURE 4:**
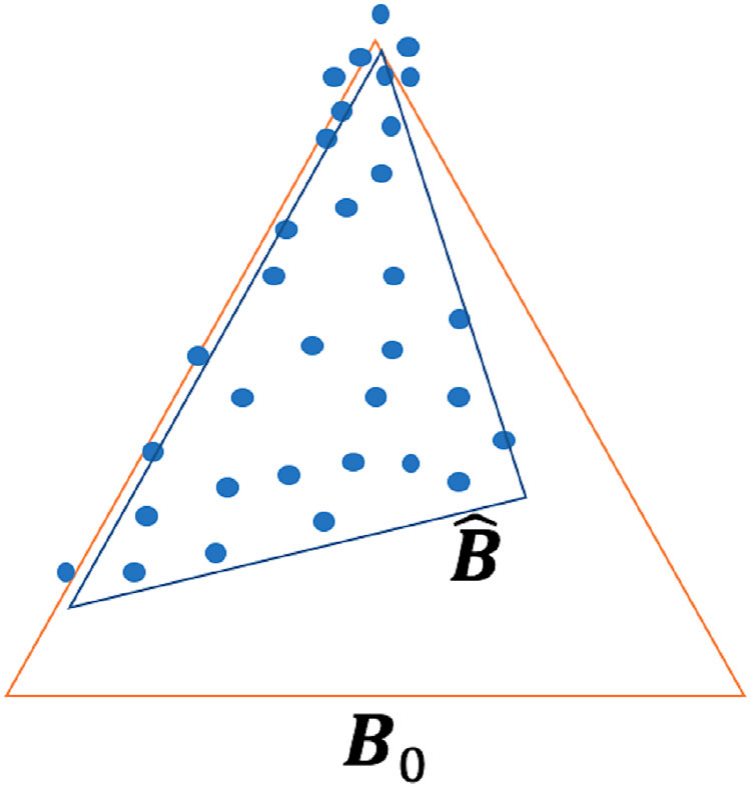
For the same three‐grain example in Figure 3, illustration of how the Stage 1 estimate $\hat{B}$ from LMM‐like algorithms is a shrunken version of $B_{0}$ when some grains have no pure regions, since $\hat{B}$ is the minimum‐volume simplex that contains the data X. In Stage 2, we recover $\hat{B_{0}}$ from $\hat{B}$, after which the columns of $\hat{B_{0}}$ can be interpreted as the DPs for the pure grains.

The SBM in Equation (3) is the same as the linear mixture model (LMM) extensively studied in the field of hyperspectral unmixing.^19^ In Stage 1 of our algorithm, we use a modified version of LMM algorithms to produce an initial estimate (denoted by $\hat{B}$) of $B_{0}$. For the situation depicted in Figure 3, in which some grains have no pure regions, $\hat{B}$ from LMM algorithms is a shrunken version of $B_{0}$, since LMM algorithms find the smallest‐volume simplex that approximately contains the data. For the same three‐grain example in Figure 3, Figure 4 depicts this difference between $\hat{B}$ and $B_{0}$. The vertices of this shrunken simplex (i.e., the columns of $\hat{B}$) do not represent DPs for the pure grains present in the nanoparticle. Consequently, we follow Stage 1 with a Stage 2 algorithm that recovers an estimate $\hat{B_{0}}$ of $B_{0}$ from $\hat{B}$, and the columns of $\hat{B_{0}}$ can then be correctly interpreted as the DPs for the pure grains.

More specifically, in Stage 1, we solve the following constrained optimisation problem with variables B, C:

(4a) $$\begin{array}{cc} \underset{B,C}{minimise}\; & ||X-{BC||}_{F}^{2}\\ & +\lambda \mathrm{Tr}\left(\left(I_{K}-\frac{1_{K}1_{K}^{T}}{K}\right)B^{T}B\left(I_{K}-\frac{1_{K}1_{K}^{T}}{K}\right)\right) \end{array}$$

(4b) $$\begin{array}{cc} \text{s.t.}\; & B\in R_{\geq 0}^{d\times K}, \end{array}$$

(4c) $$\begin{array}{cc} & C\in R_{\geq 0}^{K\times n},\;C^{T}1_{K}=1_{n}, \end{array}$$

where $\left|\right|\cdot {\left|\right|}_{F}^{2}$ denotes the square of Frobenius norm, Tr(·) denotes the trace operator, $I_{K}$ denotes the K×K identity matrix, and λ is a hyperparameter that penalises a surrogate for the simplex volume (the second term in the objective function) and balances this with the data fitting error. The membership matrix C in Stage 1 is associated with B and not $B_{0}$. This is a modified version of LMM algorithms, since LMM algorithms generally use $\log det(B^{T}B)$ as a surrogate for the simplex volume. This modification is motivated by two considerations: (i) the logarithm can yield negative values and has a global minimum of −∞ when B is singular; (ii) as a surrogate for simplex volume, the trace term that we use in Equation (4a) scales with d and K similarly to the reconstruction error, $||X-{BC||}_{F}^{2}$, which enables a simple heuristic for selecting the hyperparameter λ that we found to work well across different problem sizes (i.e., different d and K), as well as different noise levels (see Section 3.3).

Given $\hat{B}$ from Stage 1, to estimate $B_{0}$ in Stage 2, we make use of the fact that $B_{0}$ will typically be sparser (have more elements that are zero) than B, since the columns of B represent linear combinations of the columns in $B_{0}$, and the columns of $B_{0}$ represent the DPs for pure grains (which will have numerous zero entries if many of the BD locations for the pure grains differ). To estimate $B_{0}$, we adapt the varimax rotation criterion from factor analysis,^20^ which was designed to transform factor loading matrices into sparser matrices in the factor rotation setting. Specifically, our Stage 2 optimisation problem with decision variables $B_{0}$, P is

(5a) $$\begin{array}{cc} \underset{B_{0},P}{maximise}\; & Varimax(B_{0}) \end{array}$$

(5b) $$\begin{array}{cc} \text{s.t.}\; & B_{0}\in R_{\geq 0}^{d\times K}, \end{array}$$

(5c) $$\begin{array}{cc} & P\in R_{\geq 0}^{K\times K},\;P^{T}1_{K}=1_{K}, \end{array}$$

(5d) $$\begin{array}{cc} & B_{0}P=\hat{B}, \end{array}$$

with

(6a) $$\begin{array}{cc} & Varimax\left(B_{0}\right)=\frac{1}{d}\sum_{k=1}^{K}\left(\sum_{j=1}^{d}{\tilde{b}}_{j,k}^{4}-\frac{{\left(\sum_{j=1}^{d}{\tilde{b}}_{j,k}^{2}\right)}^{2}}{d}\right), \end{array}$$

(6b) $$\begin{array}{cc} & {\tilde{b}}_{j,k}=\frac{b_{0,j,k}}{h_{j}},\;h_{j}^{2}=\sum_{k=1}^{K}b_{0,j,k}^{2}. \end{array}$$

Note that the matrix P (each column of which has nonnegative elements that sum to 1) in Equation (5d) represents the extent to which $\hat{B}$ is shrunk, relative to $B_{0}$. The varimax objective in Equation (6a) sums the variance of squared normalised entries in each column of $B_{0}$. Maximising this objective encourages solutions where the rows of $B_{0}$ have nonzero elements in as few columns as possible, which is the sparsity consideration that allows $B_{0}$ to be recovered, as demonstrated in our later numerical experiments. The constraint Equation (5d) ensures that each vertex in $\hat{B}$ is contained within the simplex defined by $B_{0}$, and represents an invertible transformation between $B_{0}$ and $\hat{B}$.

Finally, we estimate the membership matrix $C_{0}$ in Equation (3) by regressing X onto the column space of $\hat{B_{0}}$, as described at the end of Section 3.2.

### 3.1 Stage 1: Optimisation algorithm

Because the objective function in Equation (4a), which we denote here by $f_{1}\left(B,C\right):=||X-{BC||}_{F}^{2}+\lambda \mathrm{Tr}\left(\left(I_{K}-\frac{1_{K}1_{K}^{T}}{K}\right)B^{T}B\left(I_{K}-\frac{1_{K}1_{K}^{T}}{K}\right)\right)$, is not jointly convex in both B and C, we solve the Stage 1 optimisation by alternatively updating B and C with adaptively chosen step sizes and projection onto the feasible constraint space as in Algorithm 1 below. In Algorithm 1, $\alpha^{B}$ and $\alpha^{C}$ denote the gradient descent step size for updating B and C, respectively; $\nabla_{B}$ and $\nabla_{C}$ denote the gradient of $f_{1}\left(B,C\right)$ w.r.t. B and C; $\Pi_{B}$ and $\Pi_{C}$ denote operators that project the updated estimates of B and C onto the constraint space defined by Equations (4b) and (4c). For the initial guesses $\hat{B}$, $\hat{C}$, we use the corresponding quantities from NMF, and we compute λ as described in Section 3.3. In each epoch, we first update B and C by taking a step in the gradient descent direction with step sizes selected adaptively via Algorithm 3 and the gradients computed via

(7a) $$\begin{array}{cc} & \nabla_{B}f_{1}=-2\left(X-BC\right)C^{T}+2\lambda B\left(I_{K}-\frac{1_{K}1_{K}^{T}}{K}\right)\\ & \left(I_{K}-\frac{1_{K}1_{K}^{T}}{K}\right), \end{array}$$

(7b) $$\begin{array}{cc} \nabla_{C}f_{1} & =-2\left(X-BC\right)B^{T}. \end{array}$$

We then project the updated $\hat{B}$ and $\hat{C}$ to their feasible constraint spaces via

(8a) $$\begin{array}{cc} \Pi_{B}\left(\hat{B}\right) & =max(\hat{B},\;0), \end{array}$$

(8b) $$\begin{array}{cc} \Pi_{C}\left(\hat{C}\right) & =argmin_{C\in R_{\geq 0}^{K\times n},\;C^{T}1_{K}=1_{n}}||C-\hat{C}{\left|\right|}_{F}^{2}, \end{array}$$

where Equation (8b) can be efficiently solved.^21^ Algorithm 1 terminates upon the convergence of the objective function, $f_{1}$.

ALGORITHM 1 **Stage 1 algorithm**

**Table:** 

**Require**: $\hat{B}$, $\hat{C}$ from NMF, and compute λ as in Equation (13).
**Require**: Choose $\alpha^{B}=0.01$, $\alpha^{C}=1$, ρ=0.5, $error_{old}=f_{1}\left(\hat{B},\hat{C}\right)$, $\varepsilon =10^{-6}$, and $Epoch_{max}=2000$.
**for** e in $\left\{1,2,\text{…},Epoch_{max}\right\}$ **do**
Obtain $\alpha^{C}$ by Algorithm 3;
$\hat{C}\leftarrow \Pi_{C}\left(\hat{C}-\alpha^{C}\nabla_{C}f_{1}\right)$;
Obtain $\alpha^{B}$ by Algorithm 3;
$\hat{B}\leftarrow \Pi_{B}\left(\hat{B}-\alpha^{B}\nabla_{B}f_{1}\right)$;
$error\leftarrow f_{1}\left(\hat{B},\hat{C}\right)$;
**if** $\frac{|error_{old}-error|}{|error_{old}|}<\varepsilon$ **then**
**Break**
**end if**
$error_{old}\leftarrow error$;
**end for**
**Return** $\hat{B}$.

### 3.2 Stage 2: Optimisation algorithm

Because the varimax objective, which we denote here by $f_{2}\left(B_{0}\right):=Varimax\left(B_{0}\right)$ is nonconvex, we apply the gradient ascent method as in the Algorithm 2 below. In Algorithm 2, the gradient of $f_{2}$ w.r.t. the row‐s, column‐t element of $B_{0}$ is calculated as

(9) $$\begin{array}{cc} \nabla_{b_{0,s,t}}f_{2}= & \frac{4{\tilde{b}}_{s,t}}{dh_{s}}\left({\tilde{b}}_{s,t}^{2}-\sum_{j=1}^{K}{\tilde{b}}_{s,j}^{4}\right)\\ & -\frac{4{\tilde{b}}_{s,t}}{d^{2}h_{s}}\left(\sum_{i=1}^{d}{\tilde{b}}_{i,t}^{2}-\sum_{j=1}^{K}\left(\sum_{i=1}^{d}{\tilde{b}}_{i,j}^{2}\right){\tilde{b}}_{s,j}^{2}\right), \end{array}$$

and the d×K matrix gradient is

(10) $$\begin{array}{cc} \nabla_{B_{0}}f_{2} & =\frac{4}{d}\left(\tilde{B_{0}}⊘H\right)⊙\left(E-\left(E⊙E\right)1_{K}1_{K}^{T}\right)\\ & -\frac{4}{d^{2}}\left(\tilde{B_{0}}⊘H\right)⊙\left(1_{d}1_{d}^{T}E-\left(\left(1_{d}1_{d}^{T}E\right)⊙E\right)1_{K}1_{K}^{T}\right),\\ E & =\tilde{B_{0}}⊙\tilde{B_{0}},\;H={\left[h_{1},\text{…},h_{d}\right]}^{T}1_{K}^{T}, \end{array}$$

where ⊙ denotes element‐wise multiplication, and ⊘ denotes element‐wise division. The matrix H is a normalisation matrix with $h_{j}\;\left(j=1,\text{…},d\right)$ computed by Equation (6b), and $\tilde{B_{0}}$ is the normalised matrix with each entry computed by Equation (6b). The operators $\Pi_{B_{0}}$ and $\Pi_{P}$ project the updated matrices $\hat{B_{0}}$, $\hat{P}$ onto the feasible constraint spaces defined by Equations (5b) and (5c), respectively, similarly to Equations (8a) and (8b), via

(11a) $$\begin{array}{cc} \Pi_{B_{0}}\left(\hat{B_{0}}\right) & =max(\hat{B_{0}},\;0), \end{array}$$

(11b) $$\begin{array}{cc} \Pi_{P}\left(\hat{P}\right) & =argmin_{P\in R_{\geq 0}^{K\times K},\;P^{T}1_{K}=1_{K}}||P-\hat{P}{\left|\right|}_{F}^{2}. \end{array}$$

Algorithm 2 solves for an optimal transformation P such that $\hat{B_{0}}:=\hat{B}P^{-1}$ maximises the varimax objective. During each epoch, we first update $\hat{B_{0}}$ by taking a step in the projected (onto the nonnegativity constraint space) gradient ascent with an adaptive step size. Then, we perform a local optimisation to iteratively refine both $\hat{B_{0}}$ and $\hat{P}$, projecting them back onto the constraint space defined by Equation (5d) that relates them. Algorithm 2 terminates when the varimax objective stabilises between successive epochs.

Given $\hat{B_{0}}$, we estimate the membership matrix $C_{0}$ via

(12) $$\begin{array}{c} \hat{C_{0}}=argmin_{C_{0}\in R_{\geq 0}^{K\times n},\;C_{0}^{T}1_{K}=1_{n}}\left|\right|C_{0}-{\left({\hat{B_{0}}}^{T}\hat{B_{0}}\right)}^{-1}{\hat{B_{0}}}^{T}X{\left|\right|}_{F}^{2}, \end{array}$$

which first regresses X onto the column space of $\hat{B_{0}}$, then projects each column of the resulting matrix, ${\left({\hat{B_{0}}}^{T}\hat{B_{0}}\right)}^{-1}{\hat{B_{0}}}^{T}X$, onto the K−1 dimensional probability simplex^21^ via the minimisation over $C_{0}$.

ALGORITHM 2 **Stage 2 algorithm**

**Table:** 

**Require**: Initial guesses, $\hat{B_{0}}=\hat{B}$, $\hat{P}=I_{K}$.
**Require**: $error_{old}=f_{2}\left(\hat{B_{0}}\right)$, $\varepsilon =10^{-6}$, $\delta =10^{-4}$, $Epoch_{max}\;=200$.
**for** e in $\left\{1,2,\text{…},Epoch_{max}\right\}$ **do**
Calculate $\nabla_{B_{0}}f_{2}$, obtain α by Algorithm 3;
$\hat{B_{0}}\leftarrow \Pi_{B_{0}}\left(\hat{B_{0}}+\alpha \nabla_{B_{0}}f_{2}\right)$;
**while** $\left|\right|\hat{B}-\hat{B_{0}}\hat{P}{\left|\right|}_{F}^{2}\geq \delta$ **do**
$\hat{P}\leftarrow \Pi_{P}\left({\left({\hat{B_{0}}}^{T}\hat{B_{0}}\right)}^{-1}{\hat{B_{0}}}^{T}\hat{B}\right)$;
$\hat{B_{0}}\leftarrow \Pi_{B_{0}}\hat{B}{\left(\hat{P}\right)}^{-1}$;
**end while**
$error\leftarrow f_{2}\left(\hat{B_{0}}\right)$;
**if** $\frac{|error_{old}-error|}{|error_{old}|}<\varepsilon$ **then**
**Break**
**end if**
$error_{old}\leftarrow error$;
**end for**
**Return** $\hat{B_{0}}$.

### 3.3 Selection of λ and adaptive step size heuristic

In Equation (4a), the hyperparameter λ balances the data fitting error against the simplex volume penalty. If λ is too large, the algorithm over‐penalises the simplex volume term, causing the simplex whose vertices are the columns of $\hat{B}$ to be too small to encompass the data points in X. Conversely, if λ is too small, the simplex may be too large.

To select λ we use the rule

(13) $$\begin{array}{c} \lambda =\theta \frac{||X-\hat{B}\hat{C}{\left|\right|}_{F}^{2}}{\mathrm{Tr}\left(\left(I_{K}-\frac{1_{K}1_{K}^{T}}{K}\right){\hat{B}}^{T}\hat{B}\left(I_{K}-\frac{1_{K}1_{K}^{T}}{K}\right)\right)}, \end{array}$$

where θ is a hyperparameter that can be selected more intuitively than λ (as discussed below), $\hat{B}$ and $\hat{C}$ are the Algorithm 1 initial guesses for B and C from NMF. The right‐hand‐side of Equation (13) represents the ratio of the first (data fitting error) term and the second (volume regularisation) term for the initial guesses in the Algorithm 1 objective function, multiplied by θ. Consequently, selecting $\theta =10^{-3}$ (which we use for all numerical simulations in Section 4) results in the second term $\lambda \mathrm{Tr}\left(\left(I_{K}-\frac{1_{K}1_{K}^{T}}{K}\right){\hat{B}}^{T}\hat{B}\left(I_{K}-\frac{1_{K}1_{K}^{T}}{K}\right)\right)$ being $\theta =10^{-3}$ times the first term $||X-\hat{B}\hat{C}{\left|\right|}_{F}^{2}$, at least for the initial guesses.

The Stage 1 and Stage 2 algorithms both require an adaptive step size α, for which we use the approach in the Algorithm 3 below. To simplify the notation, let f(z) (i.e., either $f_{1}\left(\cdot \right)$ or $-f_{2}\left(\cdot \right)$) denote the objective function to be minimised and z denote the set of decision variables. The gradient descent direction is defined as $g:=-\nabla_{z}f$, and each iteration of the optimisation algorithm takes a step αg in the gradient direction with step size α determined from Algorithm 3.

In each epoch of the Stage 1 and Stage 2 optimisation algorithms, we initialise the step size $\alpha_{old}$ for the current epoch by doubling the selected step size from the previous epoch. Then at each iteration of Algorithm 3, we reduce the step size by a specified factor ρ (we use ρ=0.5 for all simulations in Section 4) via $\alpha_{new}=\rho \alpha_{old}$. The iterations continue until the function $f(z+\alpha_{new}g)$ at the reduced step size is larger (worse) than the function $f(z+\alpha_{old}g)$ at the current step size.

ALGORITHM 3 **Adaptive step size algorithm**

**Table:** 

**Require**: step size from last epoch $\alpha_{old}$, current variable z, and update direction g.
$\alpha_{old}\leftarrow 2\alpha_{old}$
**while** $\alpha_{old}\geq 10^{-6}$ **do**
$\alpha_{new}\leftarrow \rho \alpha_{old}$
**if** $f\left(z+\alpha_{new}g\right)>f\left(z+\alpha_{old}g\right)$ **then**
**Break**
**end if**
$\alpha_{old}\leftarrow \alpha_{new}$
**end while**
**Return** $\alpha_{old}$

## 4 NUMERICAL RESULTS

**FIGURE 5:**
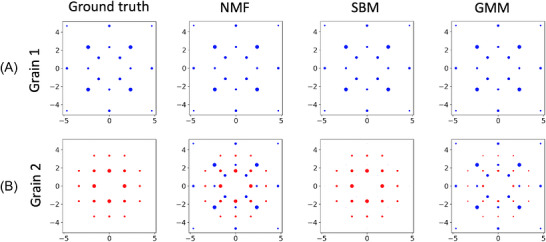
Ground truth DPs (left column) and estimated DPs from the three methods for the two‐grain noiseless data example.

**FIGURE 6:**
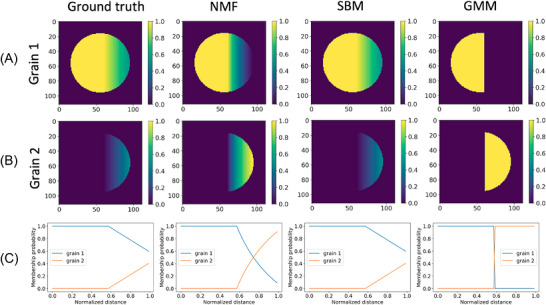
Ground truth and estimated membership heatmaps (A, B) and line plots (C) of the three methods for the noiseless two‐grain example with DPs shown in Figure 5.

To demonstrate the performance of SBM, we simulated datasets that contain two and three grains by directly simulating the data matrix X. For each example, we first consider the case that X is noiseless and then the case that X contains noise. The DPs (BD locations and intensities) for the two‐grain example are shown in the ground truth (leftmost) column of Figure 5, and their memberships at each scan position are shown in the ground truth column of Figure 6. Note that the ground truth DPs determine $B_{0}$ and the ground truth memberships determine $C_{0}$, with the data in the noiseless case being $X=B_{0}C_{0}$. For the noisy case, we introduced multiplicative intensity noise to X by replacing $x_{j,i}\leftarrow \alpha_{j,i}x_{j,i}$ with the noise $\alpha_{j,i}$ normally independently distributed with mean 1.0 and standard deviation 0.2, which represents a significant level of intensity fluctuation. In Appendix D we provide analogous simulation results in which the full 4D‐STEM data are simulated, by first simulating DPs at each scan position and then forming X via the data preprocessing pipeline described in the introduction.

For each dataset, we compare the performances of SBM, NMF and GMM^12^ across two key aspects: (i) accuracy of the estimated DPs of the pure grains, which are taken to be the columns of the estimated $\hat{B_{0}}$; (ii) accuracy of the membership maps, with the relative memberships of the grains at each scan position taken to be the corresponding column of $\hat{C_{0}}$. Regarding (i), when plotting the estimated DPs (e.g., as in Figure 5), for easier visualisation and comparison to the ground truth DP, we scale the intensities of all BDs in an estimated DP proportionally so that the BD with highest intensity has the same intensity as the corresponding ground truth BD. The rationale is that the relative intensities of the BDs in each DP are more important than the absolute intensities, in terms of identifying the crystal structure of the grain. Regarding (ii), we visualise the memberships two ways: via a heatmap showing how the memberships vary across the entire nanoparticle (e.g., as in Figures 6a and b) and via a line plot of how the memberships vary along a horizontal line segment traversing the nanoparticle from left‐to‐right at the vertical centre of the nanoparticle (e.g., as in Figure 6c). Memberships are constrained to the interval [0,1] with a membership of 1 indicating a pure grain pixel. The membership maps of NMF and SBM typically contain some level of speckled noise, so for clearer visualisation we applied a median pass filter to spatially smooth the maps before plotting, as described in Appendix C. Implementation details for NMF and GMM are provided in Appendix A. The computation time for the SBM algorithm is reported in Appendix E.

### 4.1 Two‐grain nanoparticle example, noiseless X

We consider the same two‐grain nanoparticle depicted in Figure 3a. The region to the left of the red dashed line consists of pure crystal grain 1, while the right is an overlap of grains 1 and 2. Notably, grain 2 lacks a pure region and has maximum membership of only around 0.5 (at the right edge of the nanoparticle), which makes it challenging to estimate its pure DP. More detailed ground truth membership maps for both grains are provided in the ground truth column of Figure 6.

The ground truth DPs for the two grains are shown in Figure 5, as well as the estimated DPs for the three methods. To more easily distinguish which BDs are from grain 1 versus grain 2 in the estimated DPs, we have colour coded the BDs according to their ground truth grains. This is only for easier comparison of the results, since this information would not be available in practice. All three methods are able to estimate the DP for grain 1 reasonably well. However, for the DP of grain 2, both NMF and GMM estimate it to be a mixture, while SBM provides a much more accurate estimate that represents the pure DP for grain 2.

SBM achieves nearly perfect recovery of the ground truth membership, as evident in both the heatmaps (Figure 6a and b) and membership line plots (Figure 6c). While NMF performs reasonably well, it significantly overestimates the membership of grain 2. Based on the membership line plot in Figure 6c, NMF estimates grain 2 membership exceeding 0.8 at the rightmost end of the path. GMM is less effective than the other two methods, as it concludes the transition from grain 1 to grain 2 is abrupt with almost no overlapping region. This behaviour is not unexpected, since GMM is designed to assign data points to discrete clusters rather than accurately infer fractional memberships. Nevertheless, GMM successfully differentiates the pure region of crystal 1 from the overlapped region. The more accurately estimated membership maps and DPs produced by SBM imply that it would allow a more accurate 3D reconstruction of the entire nanoparticle.

### 4.2 Two‐grain nanoparticle example, noisy X

**FIGURE 7:**
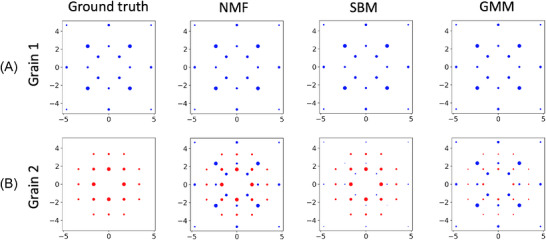
Ground truth DPs (left column) and estimated DPs from the three methods for the two‐grain noisy data example.

**FIGURE 8:**
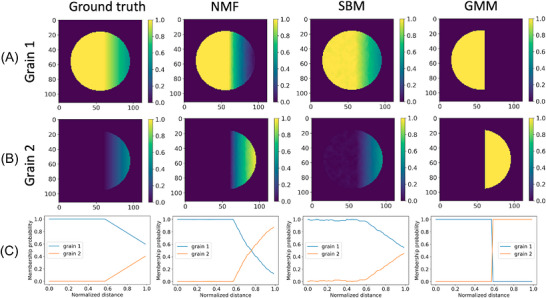
Ground truth and estimated membership heatmaps (A, B) and line plots (C) of the three methods for the noisy two‐grain example with DPs shown in Figure 7.

Analogous results for the noisy version of X are shown in Figure 7 (estimated DPs) and Figure 8 (estimated memberships). Again, all three methods accurately recover DP1 due to the presence of its pure region, but they estimate DP2 less accurately. The DP2 estimate from NMF appears as a balanced mixture of the ground truth DP1 and DP2, whereas the GMM estimate of DP2 is closer to the ground truth DP1 than DP2. The estimated membership maps are shown in Figure 8. As for the noiseless case, SBM again estimates the membership maps much more accurately than NMF or GMM.

### 4.3 Three‐grain nanoparticle example, noiseless X

We consider the same three‐grain nanoparticle depicted in Figure 3b: the leftmost region consists of pure grain 1, the middle region contains a mixture of grain 1 and grain 2, and the remaining area is a complex mixture of all three grains. The ground truth membership maps shown in Figure 10 underscore the challenge: neither grain 2 nor grain 3 possesses a pure region. In particular, the maximum membership is approximately 0.7 for grain 2 and only around 0.5 for grain 3.

**FIGURE 9:**
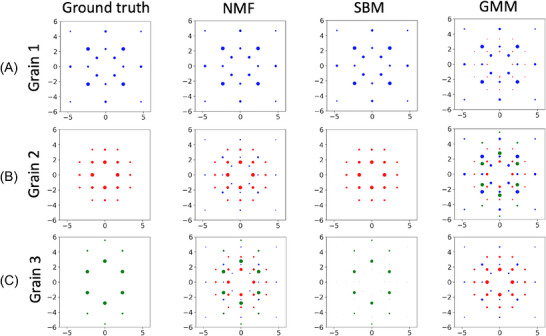
Ground truth DPs (left column) and estimated DPs from the three methods for the three‐grain noiseless data example.

**FIGURE 10:**
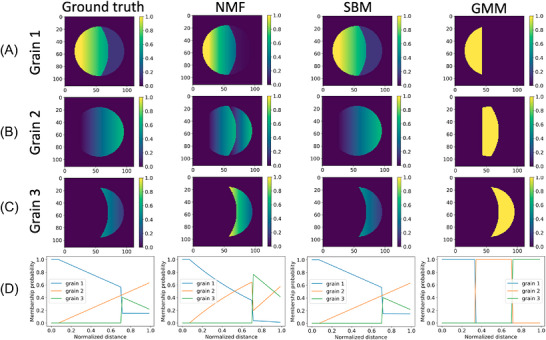
Ground truth and estimated membership heatmaps (A, B, C) and line plots (D) of the three methods for the noiseless three‐grain example with DPs shown in Figure 9.

Even in this noiseless case, NMF and GMM struggle to recover the ground truth DPs as shown in Figure 9. NMF estimates DP2 to be a mixture of ground truth DP1 and DP2, and its DP3 estimate is a composite of all three DPs. GMM estimates DP2 as a three‐pattern mixture and DP3 as a combination of DP1 and DP2. On the other hand, SBM estimates DP2 and DP3 nearly perfectly, with the estimated DP3 containing only very small intensities for BDs of grains 1 and 2.

SBM also estimates the membership maps nearly perfectly, including detecting the small membership of grain 1 in the rightmost region of the nanoparticle, as shown in Figure 10. In contrast, NMF inflates the membership of grain 2 in the middle region and the membership of grain 3 in the right region in which all three grains overlap. GMM fails to identify any overlapping regions and concludes that all transition between the three grains are abrupt, which is consistent with its estimated DPs in Figure 9 all being mixtures of the ground truth DPs.

### 4.4 Three‐grain nanoparticle example, noisy X

**FIGURE 11:**
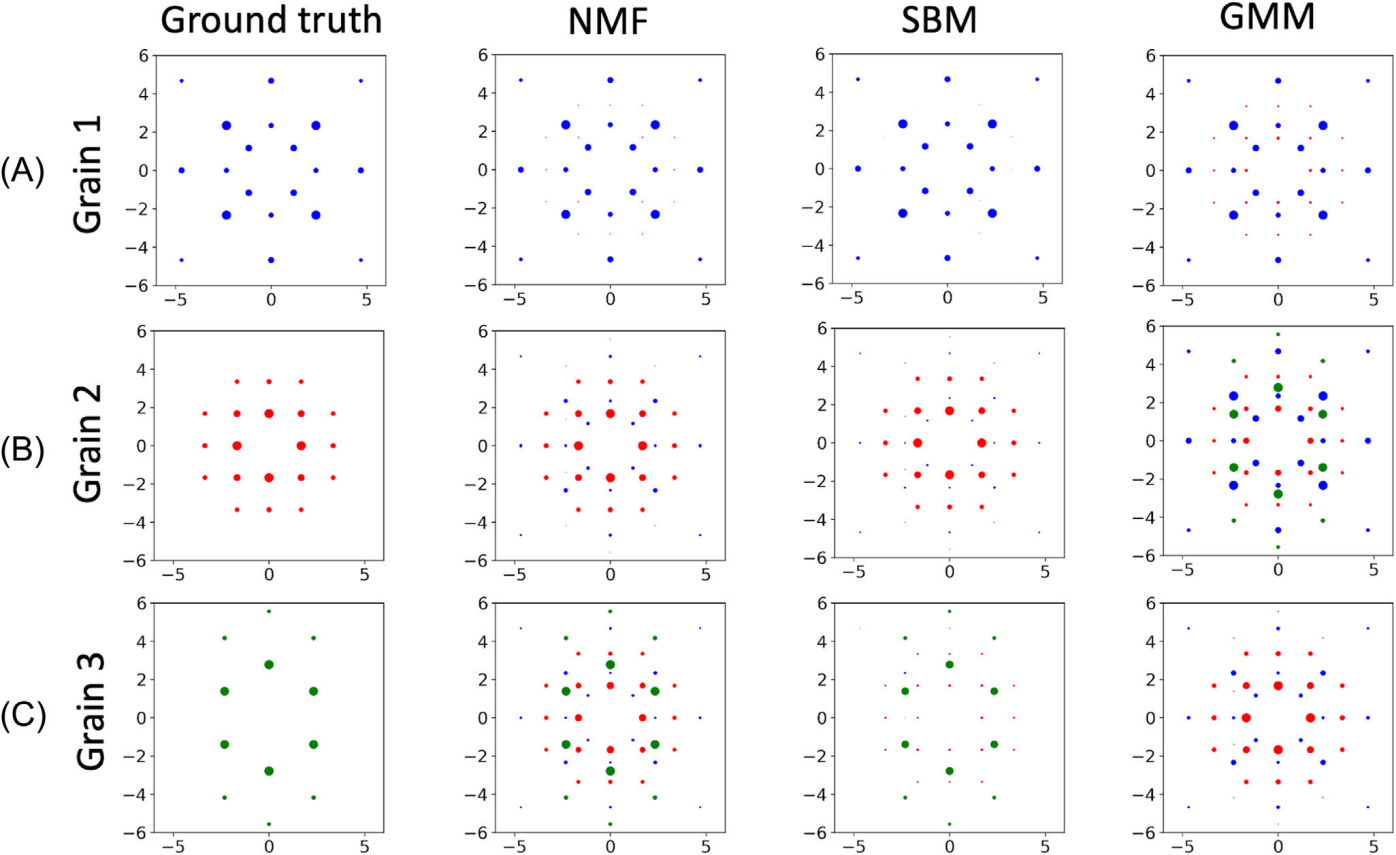
Ground truth DPs (left column) and estimated DPs from the three methods for the three‐grain noisy data example.

**FIGURE 12:**
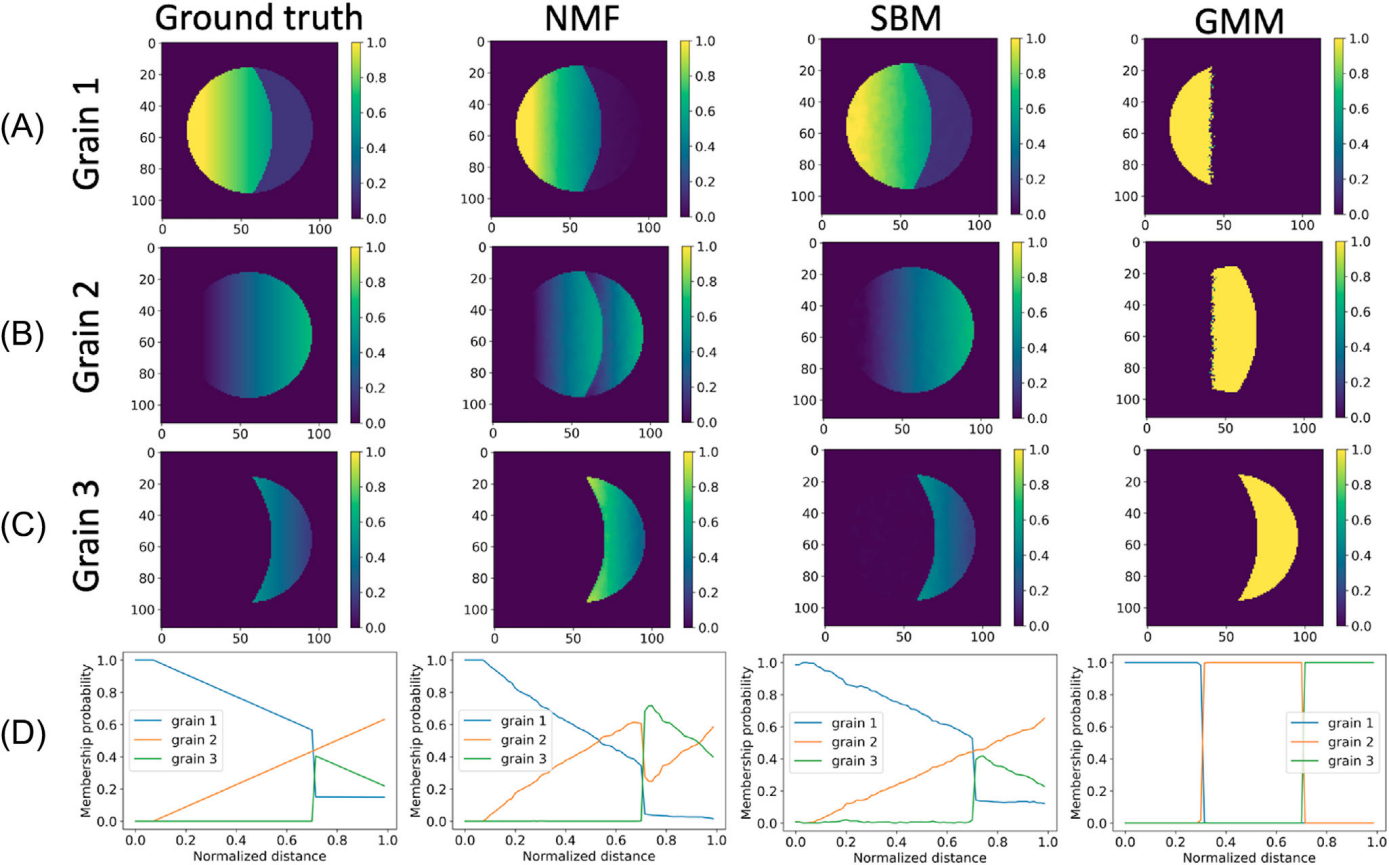
Ground truth and estimated membership heatmaps (A, B, C) and line plots (D) of the three methods for the noisy three‐grain example with DPs shown in Figure 11.

When noise is added, the estimated DPs from all three methods are less accurate, although the SBM estimates remain substantially more accurate than for the other two methods (Figure 11). Similarly, Figure 12 shows that the estimated membership maps are much more accurate for SBM than for NMF and GMM.

## 5 CONCLUSIONS

This paper introduces a simplex‐based model (SBM) to represent nanoparticle 4D‐STEM data and presents a SBM‐based algorithm for accurately estimating the pure‐grain DPs and spatial membership maps. Numerical results for simulated nanoparticles with various configurations and noise levels demonstrate that the approach can successfully estimate the structure of the nanoparticle even in challenging scenarios when some grains lack a pure region. The success of SBM stems from two key factors: (i) the simplex constraint ensures that estimated DPs remain physically plausible convex combinations, and (ii) the varimax‐based sparsity promotion in Stage 2 drives the estimated DPs toward pure‐grain patterns even when no pure pixels exist in the data.

One limitation of the approach is that the method depends on the extracted feature matrix X, which is inherently influenced by the BD detection accuracy. In practice, peak detection errors, overlapping disks, and intensity distortions can propagate into X. For example, if there is substantial overlap in the BDs for the pure grains present in the nanoparticle, the grains will be difficult to separate, but it is unlikely that any method will be able to separate the grains in this case. As a proof of concept, we provide simulations considering noise when simulating X and detection errors when simulating the 4D‐STEM dataset. We intend to conduct further investigations over experimental datasets to validate the method under the more complex perturbations as discussed above. Another limitation lies in the assumption that the DPs are convex combinations of pure patterns, implying that BD intensities behave approximately linearly. This assumption is reasonable for overlapping grains in thin samples, but might be compromised by dynamical diffraction, strain, or thickness variations. While our simulations demonstrate that the method is robust to significant random noise, it may not be directly applicable if these physical factors affect X in a more systematic manner.

The estimation results produced by the SBM algorithm provide a nearly complete set of information for creating a 3D reconstruction of the entire nanoparticle. This information consists of (i) the 2D spatial locations and geometry/shape of each grain; (ii) a membership map that represents the relative presence of each grain at each scan position in the nanoparticle; and (iii) a pure grain DP for each grain. Producing pure grain DPs with the SBM algorithm, rather than estimated DPs that are mixtures of the pure DPs (as often observed with NMF and GMM in our numerical examples), greatly simplifies identification of the crystal structure of each grain. This can be achieved either through human inspection of the DPs and/or via an automated downstream classification algorithm, such as a neural network classifier.^22^

The primary missing piece of information for creating a 3D reconstruction is the vertical (perpendicular to the 2D scan plane) ordering of the grains at scan positions where multiple grains overlap, since the SBM algorithm only provides the relative memberships of the grains at each position and not their ordering. One potential solution is to combine the SBM results with depth sectioning that utilises aberration‐corrected STEM to analyse the depth sensitivity at nanoscale,^23^ which is a promising avenue of future work.

## AUTHOR CONTRIBUTIONS

**Wei Liu**: Conceptualisation (equal); Formal analysis (equal); Investigation (equal); Methodology (equal); Software (equal); Validation (equal); Visualisation (equal); Writing – original draft (equal). **Roberto dos Reis**: Resources (supporting); Validation (equal); Writing – review & editing (equal). **Chad A. Mirkin**: Funding acquisition (equal); Project administration (equal); Resources (equal); Supervision (equal); Writing – review & editing (equal). **Vinayak P. Dravid**: Funding acquisition (equal); Project administration (equal); Resources (equal); Supervision (equal); Writing – review & editing (equal). **Wei Chen**: Funding acquisition (equal); Project administration (equal); Resources (equal); Supervision (equal); Writing – review & editing (equal). **Daniel W. Apley**: Conceptualisation (equal); Funding acquisition (equal); Methodology (equal); Project administration (equal); Resources (equal); Supervision (equal); Writing – review & editing (equal).

## CONFLICT OF INTEREST STATEMENT

Chad A. Mirkin has financial interests in Mattiq, Inc. which could potentially benefit from the outcomes of this research. Northwestern University has financial interests relative to intellectual property related to this research. As a result of these interests, Northwestern University could ultimately potentially benefit financially from the outcomes of this research.

## Figures and Tables

**FIGURE B1:**
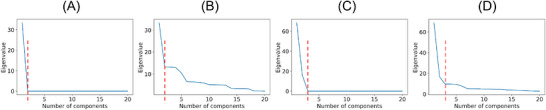
Scree plots to select K for the four examples in Section 4: (A) noiseless two‐grain nanoparticle dataset with K=2; (B) noisy two‐grain nanoparticle dataset with K=2; (C) noiseless three‐grain nanoparticle dataset with K=3; (D) noisy three‐grain nanoparticle dataset with K=3.

**FIGURE C1:**
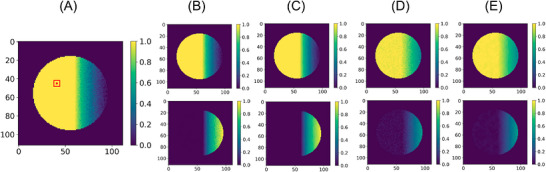
Visualisation of a median pass filter (A) and comparison between membership maps before and after the smoothing for NMF (B before, C after) and SBM (D before, E after) for the example in Figure 8.

**FIGURE D1:**
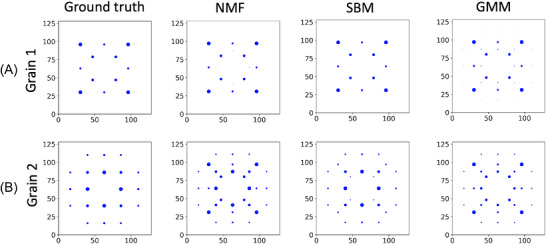
Ground truth DPs (left column) and estimated DPs from the three methods for the two‐grain noiseless data example.

**FIGURE D2:**
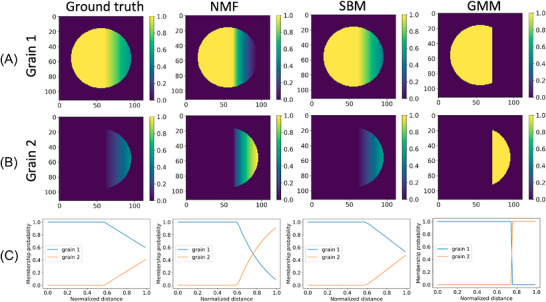
Ground truth and estimated membership heatmaps (A, B) and line plots (C) of the three methods for the noiseless two‐grain example with DPs shown in Figure D1.

**FIGURE D3:**
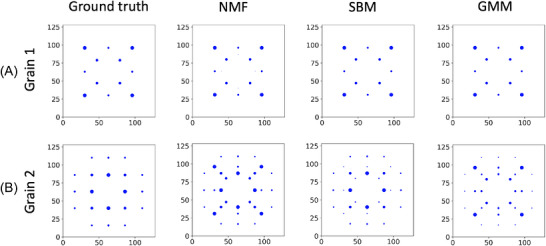
Ground truth DPs (left column) and estimated DPs from the three methods for the two‐grain noisy data example.

**FIGURE D4:**
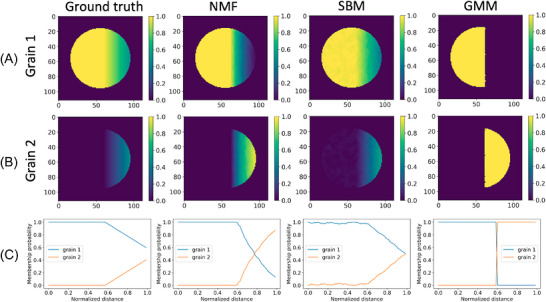
Ground truth and estimated membership heatmaps (A, B) and line plots (C) of the three methods for the noisy two‐grain example with DPs shown in Figure D3.

**FIGURE D5:**
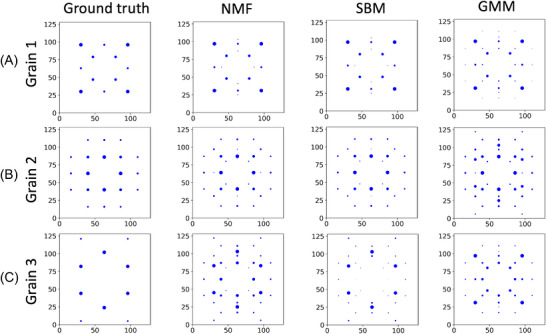
Ground truth DPs (left column) and estimated DPs from the three methods for the three‐grain noiseless data example.

**FIGURE D6:**
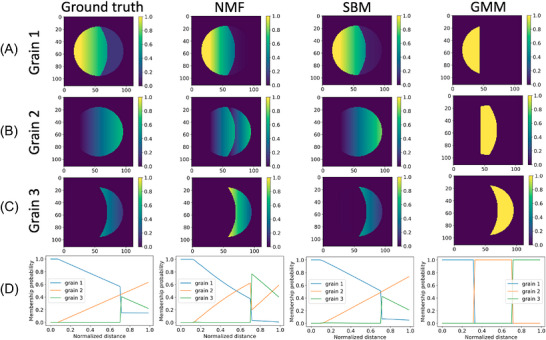
Ground truth and estimated membership heatmaps (A, B, C) and line plots (D) of the three methods for the noiseless three‐grain example with DPs shown in Figure D5.

**FIGURE D7:**
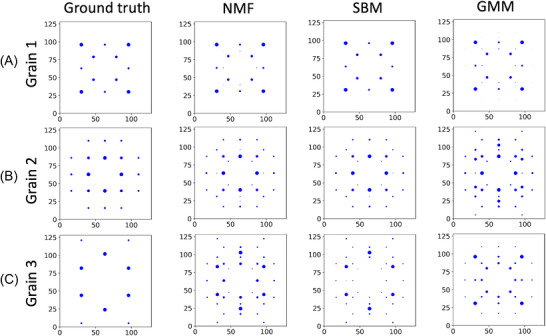
Ground truth DPs (left column) and estimated DPs from the three methods for the three‐grain noisy data example.

**FIGURE D8:**
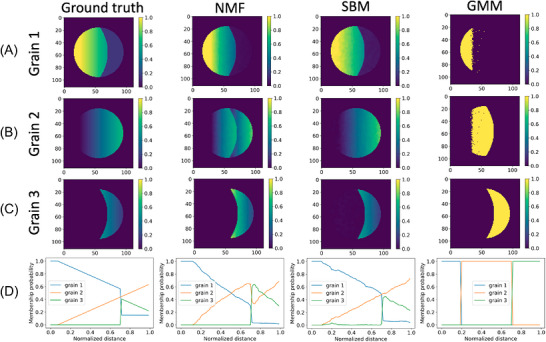
Ground truth and estimated membership heatmaps (A, B, C) and line plots (D) of the three methods for the noisy three‐grain example with DPs shown in Figure D7.

## Data Availability

The data of three example nanoparticles and code is available at https://github.com/EurusWei/SimplexBasedModelForGrainIdentification

## APPENDIX A SOME IMPLEMENTATION DETAILS FOR NMF AND GMM

NMF^24^ is a popular technique that factors a data matrix into low‐rank latent factor matrices with nonnegativity constraints. NMF model structure is

(A1)
$$\begin{array}{c} X=WH+\varepsilon ,\;\text{s.t.}\;W⪰0,\;H⪰0, \end{array}$$

where $W=\left(w_{1},\text{…},w_{K}\right)\in R^{d\times K}$ is the feature matrix, $H=\left(h_{1},\text{…},h_{n}\right)\in R^{K\times n}$ is the coefficient matrix, $\varepsilon \in R^{d\times n}$ is the error matrix, K is the rank of W and suggests the number of distinct grains in the context of 4D‐STEM data. W and H are analogous to $B_{0}$ and $C_{0}$, respectively, in our SBM Equation (3a). Two key distinctions between SBM and NMF are that NMF estimates W and H given K by minimising only the reconstruction error $||X-{WH||}_{F}^{2}$, and the elements of each column of H are not constrained to sum to one (hence it does not represent a simplex structure). In NMF, the estimated DPs are taken to be the columns of $\hat{W}$. Regarding the NMF membership maps, because the elements of the columns of $\hat{H}$ are not constrained to sum to one, we use a postprocessing step that normalises each column of $\hat{H}$ so that it sums to one and represents membership. Specifically, we take the memberships to be the elements of $\hat{H}⊘\left(1_{K}1_{K}^{T}\hat{H}\right)$.

GMM models the marginal distribution of $x_{i}$ as

(A2)
$$p\left(x_{i}\right)=\sum_{k=1}^{K}\alpha_{k}N\left(x_{i}|\mu_{k},\Sigma_{k}\right),$$

where $\alpha_{k}$ represents the prior probability that a randomly chosen observation belongs to kth segment with $\sum_{k=1}^{K}\alpha_{k}=1$. ${\left\{\alpha_{k},\mu_{k},\Sigma_{k}\right\}}_{k=1}^{K}$ are parameters of the GMM that are estimated when the GMM is fitted to the data. Let $\pi_{i}={\left[\pi_{i,1},\text{…},\pi_{i,K}\right]}^{T}$ denote the posterior probability vector, where $\pi_{i,k}$ is the posterior probability that $x_{i}$ belongs to the kth segment. GMM computes ${\left\{\pi_{i}\right\}}_{i=1}^{n}$ based on the estimates of ${\left\{\alpha_{k},\mu_{k},\Sigma_{k}\right\}}_{k=1}^{K}$.^12^

We also follow the approach^12^ and take the input to the GMM to be $\tilde{X}=\left(X_{real},\beta X^{T}\right)$, where $X_{real}$ is the real space coordinate matrix for scan positions, $\beta =10^{-4}$. The estimated DP for the kth grain is reconstructed from ${\hat{\mu }}_{k}$ by excluding its real space coordinates. The membership maps are plotted using ${\left\{{\hat{\pi }}_{i}\right\}}_{i=1}^{n}$, which inherently satisfies the sum‐to‐one constraint since ${\hat{\pi }}_{i}$ represents a posterior probability vector.

## APPENDIX B PCA SCREE PLOTS

Figure B1 shows the PCA scree plots (which plot the PCA eigenvalues in descending order) for the four nanoparticles in Section 4. We recommend selecting K to coincide with the most prominent elbow in the scree plot, which is the standard technique for selecting the number of dominant components in PCA.^25^ Specifically, if K grains are present in the nanoparticle, the simplex on which the data approximately lie (aside from noise) is K−1 dimensional, so the number of dominant eigenvalues is K−1, and elbow (just to the right of the last dominant eigenvalue) represents K. The most prominent elbows in the scree plots in Figure B1 are indicated by vertical lines. For the four examples in Figure B1, the estimated K values coincide with the true values, although the elbow is somewhat more ambiguous in Figure B1b.

## APPENDIX C MEDIAN PASS FILTER TO SMOOTH THE MEMBERSHIP MAPS

For noisy datasets, the resulting membership maps typically contain speckled noise. Consequently, before plotting the heatmaps, we postprocess the maps using the median pass filter as illustrated in Figure C1a: for each scan position (indicated by the red dot), we take its membership to be the median of membership values for neighbouring scan positions within a 5×5 pixel window (the red square) centred around that scan position. As Figure C1b–e shows, the median pass filter substantially reduces noise and improves visualisation.

## APPENDIX D NUMERICAL RESULTS FOR SIMULATED 4D‐STEM DATASETS

In Section 4, we compared the methods in examples in which we simulated X directly. In this appendix, we provide analogous numerical comparison results but with the 4D‐STEM DPs at each scan position directly simulated. In the simulated DP, we create BDs by placing an exponential kernel of the form $A_{j,i}e^{-{\left(distance^{2}/18\right)}^{4}}$ at the BD centre, restricted to a radius of distance≤4 pixels, and $A_{j,i}$ represents the intensity of the jth BD in the simulated DP at the ith scan position. Each $A_{j,i}$ is determined by the intensity of the jth BD in the pure grain DP and the grain membership at the ith scan position.

Figures D1, D2, D3, D4, D5, D6, D7, D8 present counterparts to Figures 5, 6, 7, 8, 9, 10, 11, 12 when X is distilled from the simulated 4D‐STEM DPs. Consequently, the distilled data X embodies errors from the cross‐correlation BD detection algorithm. As in Section 4, we consider two versions of each example: The ‘noiseless’ version only includes the noise from the BD detection errors. The ‘noisy’ version includes additional random noise by multiplying each BD intensity $A_{j,i}$ by a normally distributed random variable with mean 1.0 and standard deviation 0.2. A different noise variable is generated for each BD. The estimated DPs and membership maps for these examples are shown in Figures D1, D2, D3, D4, D5, D6, D7, D8, which are interpreted the same as Figures 5, 6, 7, 8, 9, 10, 11, 12 in Section 4. In the DP estimation results shown in Figures D1, D2, D3, D4, D5, D6, D7, D8, we can no longer colour code the BDs based on which pure grain they are associated with, since an individual BD in the BVM is sometimes associated with multiple grains due to the BD detection errors.

The accuracy of all methods degrades to some extent with the additional BD detection error present. However, the results are qualitatively similar to the results in Figures 5, 6, 7, 8, 9, 10, 11, 12, in that SBM provides more accurate estimation of the pure grain DPs and the membership maps in all examples. Moreover, even though the estimated DPs are somewhat less accurate than in Figures 5, 6, 7, 8, 9, 10, 11, 12, the estimated membership maps produced by SBM are still nearly perfect.

## APPENDIX E COMPUTATION TIME FOR THE SBM ALGORITHM

Table E1 presents the computation time for the SBM algorithm across all numerical examples. Note that Stage 1 dominates the computation time, primarily due to the alternative updates of B and C.

**TABLE E1:** Computation time for the SBM algorithm (unit: seconds).

condition	Simulate X		Simulate 4D‐STEM dataset	
	Noiseless	Noisy	Noiseless	Noisy
Two‐grain	126.4	5.9	4.3	17.8
Three‐grain	217.3	12.3	56.1	10.0

The computation time measures the interval from the input X into the SBM algorithm to the outputs; it excludes the data preprocessing stage for 4D‐STEM datasets.
